# Combination treatment with RAF/MEK inhibitor avutometinib and FAK inhibitor defactinib in preclinical models of meningioma

**DOI:** 10.1093/noajnl/vdag210

**Published:** 2026-08-12

**Authors:** Nazanin Ijad, Katie L Waller, Erika Yamazawa, Naema Nayyar, Danielle Burgenske, Consuelo Torrini, Brett L Carlson, Elizabeth J Summers, Christian Migliarese, Braxton Marion, Britney Shela Zhang, Emily Sullivan, Claudia Ellington, Varun Sasisekharan, Paul A Decker, Matthew Kosel, Jeanette E Eckel-Passow, Evanthia Galanis, Jann N Sarkaria, Hiroaki Wakimoto, Rachael A Vaubel, Priscilla K Brastianos

**Affiliations:** Center for Cancer Research, Massachusetts General Hospital Cancer Center, Harvard Medical School, Boston; Department of Laboratory Medicine and Pathology, Mayo Clinic, Rochester; Center for Cancer Research, Massachusetts General Hospital Cancer Center, Harvard Medical School, Boston; Center for Cancer Research, Massachusetts General Hospital Cancer Center, Harvard Medical School, Boston; Department of Radiation Oncology, Mayo Clinic; Center for Cancer Research, Massachusetts General Hospital Cancer Center, Harvard Medical School, Boston; Department of Radiation Oncology, Mayo Clinic; Center for Cancer Research, Massachusetts General Hospital Cancer Center, Harvard Medical School, Boston; Department of Neurosurgery, Massachusetts General Hospital, Harvard Medical School, Boston (C.M., H.W.); Center for Cancer Research, Massachusetts General Hospital Cancer Center, Harvard Medical School, Boston; Center for Cancer Research, Massachusetts General Hospital Cancer Center, Harvard Medical School, Boston; Center for Cancer Research, Massachusetts General Hospital Cancer Center, Harvard Medical School, Boston; Center for Cancer Research, Massachusetts General Hospital Cancer Center, Harvard Medical School, Boston; Center for Cancer Research, Massachusetts General Hospital Cancer Center, Harvard Medical School, Boston; Department of Quantitative Health Sciences, Mayo Clinic, Rochester; Department of Quantitative Health Sciences, Mayo Clinic, Rochester; Department of Quantitative Health Sciences, Mayo Clinic, Rochester; Department of Radiation Oncology, Mayo Clinic; Department of Radiation Oncology, Mayo Clinic; Department of Neurosurgery, Massachusetts General Hospital, Harvard Medical School, Boston (C.M., H.W.); Department of Laboratory Medicine and Pathology, Mayo Clinic, Rochester; Center for Cancer Research, Massachusetts General Hospital Cancer Center, Harvard Medical School, Boston

**Keywords:** BRAF inhibitor, combination treatment, FAK inhibitor, MEK inhibitor, meningioma

## Abstract

**Background:**

Meningiomas lack effective therapies for recurrent or aggressive disease. NF2 inactivation, commonly in meningioma, may sensitize tumors to FAK inhibition and interact with MAPK signaling. This study investigated the therapeutic activity of defactinib (FAK inhibitor) and avutometinib (RAF/MEK inhibitor) alone and in combination in preclinical meningioma models.

**Methods:**

MAPK-altered meningioma cell lines, IOMM-Lee (*NF2* wild-type, BRAF p.V600E) and CH157-MN (*NF2* mutant, NRAS p.Q61K), BenMen-1 (*NF2* mutant, MAPK wild-type), and three patient-derived *NF2* mutant cell lines were cultured in matrigel and treated with defactinib and avutometinib in 3D cell viability assays. *In vivo*, flank and intracranial xenograft models of IOMM-Lee and CH157-MN were treated with VS-4718 (FAK inhibitor, superior pharmokinetics in mice), avutometinib, or combination. Tumor growth, survival, and pharmacokinetic/pharmacodynamic properties were assessed.

**Results:**

*In vitro*, combination therapy showed synergistic or additive effects in CH157-MN and IOMM-Lee cells, respectively. Defactinib alone demonstrated activity in NF2-mutant patient-derived and BenMen-1 cells, while avutometinib did not. *In vivo*, avutometinib monotherapy suppressed tumor growth and extended survival in both flank and intracranial xenograft models, with evidence of favorable pharmacokinetic properties and target pathway inhibition in intracranial tumors. Combination therapy provided enhanced and sustained control of flank CH157-MN meningioma compared to avutometinib alone but did not significantly improve survival over avutometinib alone in intracranial models.

**Conclusions:**

Avutometinib demonstrated robust anti-tumor effects in flank and intracranial models of MAPK-altered meningioma. Combination benefits were context dependent. Defactinib activity in *NF2*-mutant patient-derived cells without targetable MAPK-kinase alterations highlights the importance of genomic biomarkers to guide personalized strategies.

Key Points

- Avutometinib alone and combination with VS-4718 showed efficacy in MAPK-altered meningioma xenografts.
- NF2-mutant patient-derived meningioma cells responded to defactinib but not avutometinib *in vitro*.

Importance of the Study

Meningiomas pose a significant clinical challenge due to limited treatment options beyond surgery and radiation therapy, underscoring the need for novel therapeutic strategies for recurrent or treatment-resistant disease. This study evaluated the combination of defactinib (a FAK inhibitor) and avutometinib (a RAF/MEK inhibitor) as a potential treatment for meningiomas. *In vitro*, the combination treatment synergistically or additively reduced cell survival in two MAPK-altered meningioma models, the *NF2*-mutant CH157-MN and the *NF2*-wild type IOMM-Lee cells, respectively. However, in *NF2*-mutant patient-derived meningioma cells, defactinib, not avutometinib, showed activity. *In vivo*, avutometinib monotherapy effectively suppressed tumor growth in both flank and intracranial models, exhibiting favorable pharmacokinetics in plasma and intracranial tumors. Combination therapy provided benefits in the CH157-MN flank model. These findings identify avutometinib, alone or in combination with FAK inhibition, as a promising therapeutic approach for MAPK-driven meningiomas and support further investigation, including clinical trials. Distinct responses found across the models highlight the need for genomic biomarkers to guide targeted treatments.

Meningiomas are the most common primary intracranial tumors, accounting for approximately 37% of all brain tumors.^1^ While most meningiomas are classified as benign (WHO grade 1), aggressive clinical behavior, including recurrence and treatment resistance, is common in higher grade (grade 2 and 3) meningiomas, with long term recurrence rates for grade 2 tumors exceeding 50% at 10 years and reaching nearly 80% at 15 years.^2^^,^^3^ Notably, recurrence is not limited to high-grade tumors, as even WHO grade 1 meningiomas can recur following surgery or radiotherapy.^4^ For patients with recurrent or inoperable meningiomas that have failed radiotherapy, effective pharmacological options remain limited. There is a critical unmet need in the management of this subset of refractory meningioma.

Efforts to develop effective targeted therapies for meningioma have been limited by an incomplete understanding of the signaling pathways that drive tumor growth and therapeutic resistance. However, advancements in the genetic understanding of meningiomas have identified key driver mutations such as *NF2*, *SMO*, *TRAF7*, *KLF4*, and *POLR2A*, opening avenues for genomically guided targeted therapies.^5^ Beyond DNA mutations, integrated molecular classification systems based on DNA methylation profiling, copy number alterations, and gene expression have also significantly advanced meningioma risk stratification and treatment response.^6^^,^^7^ Among these identified genomic drivers, nearly half of meningiomas harbor alteration of the tumor suppressor gene *NF2* (Neurofibromatosis type 2), which is enriched in higher-grade meningiomas and has not been previously exploited therapeutically.

Preclinical studies have identified a synthetic lethal relationship between *NF2* loss and FAK (focal adhesion kinase) inhibition, suggesting that *NF2*-deficient tumors may be particularly sensitive to FAK-targeted therapies.^8^ A recent Phase II clinical trial (NCI-MATCH ECOG-ACRIN Trial EAY131) evaluated FAK inhibitor defactinib in *NF2*-deficient tumors. This trial highlighted the potential vulnerability of these tumors to FAK inhibition, with 1 of 7 meningiomas showing partial response (PR).^9^ A genomically driven trial testing the FAK inhibitor GSK2256098 in *NF2* mutant meningiomas met primary endpoint, showing a 6-month progression-free survival of 83% and 33% in WHO grade 1 and grade 2-3 tumors, respectively.^10^ However, as the response rate was limited in both studies, there is a need to identify combination strategies to improve upon single agent FAK inhibition, both in NF2-altered and other genomically defined meningioma subsets.

In parallel, growing evidence points to dysregulated MAPK pathway activation as a critical mediator of meningioma progression, as the *NF2* encoded protein, merlin, plays a role as a key upstream suppressor of the MAPK signaling pathway (Figure 1A).^11^ Early studies identified increased MAPK signaling in higher-grade and recurrent meningiomas, with elevated ERK phosphorylation (pERK) observed in tumor specimens compared to normal meninges.^12^^,^^13^ MEK inhibition has been investigated in preclinical models of meningioma.^14^ Crosstalk between FAK and MAPK signaling has been suggested by work showing FAK inhibition induced an increase of pERK, and conversely, MAPK inhibition induced activation of FAK signaling in melanoma, contributing to resistance to single pathway inhibition.^15^^,^^16^ To address this, Paradis et al. identified synthetic lethality between FAK and MEK inhibitors in melanoma models.^17^ The combination of FAK and MEK inhibitors has been tested in *NF2*-deficient mesothelioma,^18^ providing a strong rationale for a dual-target approach. Inspired by such research in other malignancies, we hypothesized that targeting these 2 pathways simultaneously could enhance therapeutic efficacy and overcome resistance in meningiomas.

**Figure 1.**
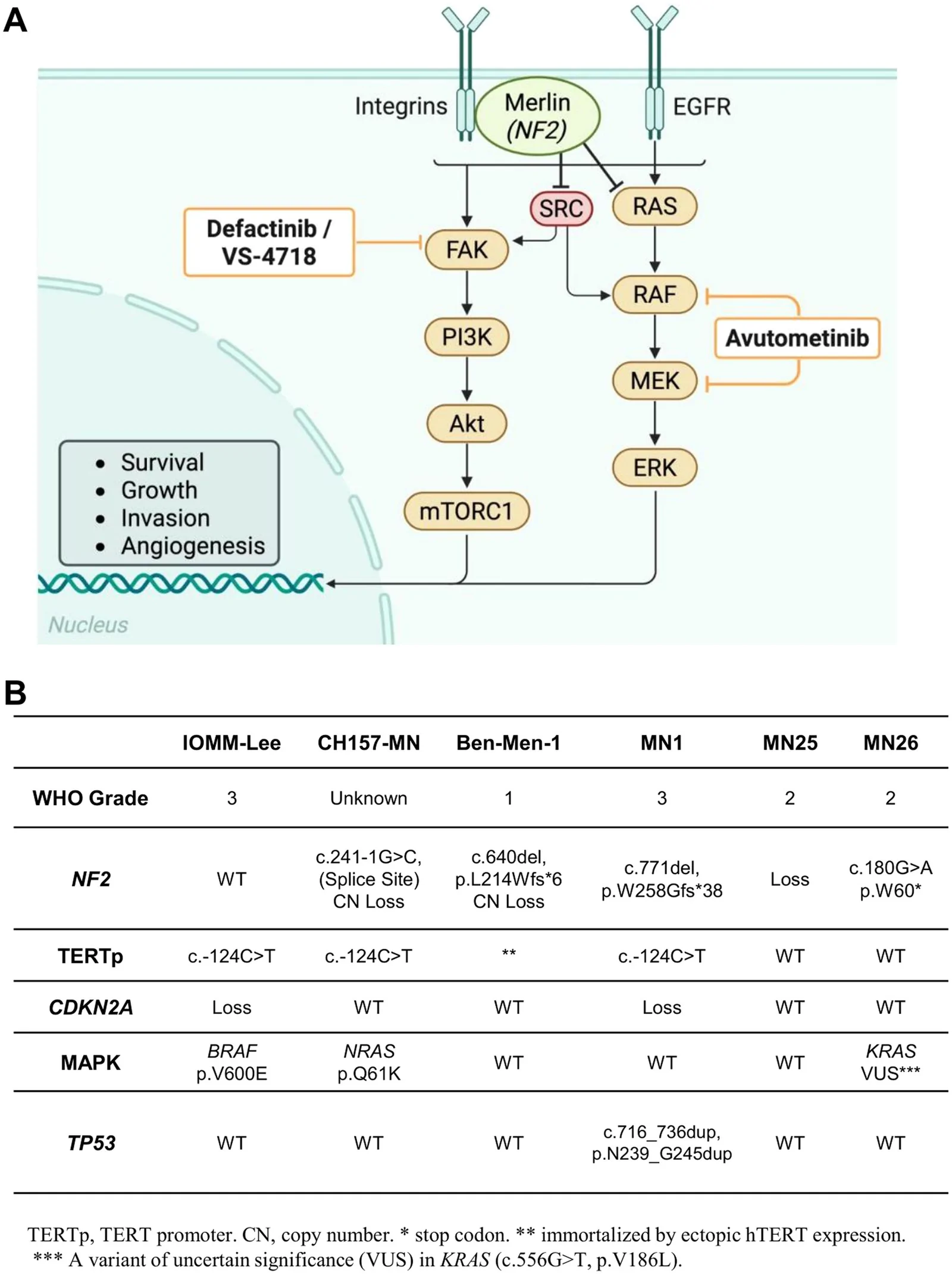
Therapeutic targets and meningioma cell line models used in this study. (A) Schematic representation of the FAK and MAPK signaling pathways. NF2 functions as a tumor suppressor regulating signaling through integrins and receptor tyrosine kinases such as EGFR. Loss of NF2 results in activation of FAK and downstream PI3K/Akt/mTOR signaling. The RAF/MEK/ERK pathway is also activated via RAS mutations or loss of NF2-mediated inhibition. FAK inhibitors (Defactinib, VS-4718) and Avutometinib target these pathways to suppress tumor growth, invasion, and angiogenesis. (B) Genetic and molecular characteristics of conventional and patient-derived meningioma cell lines used in this study. CH157-MN harbors an *NF2* splice-site mutation and loss, along with an *NRAS* Q61K mutation, whereas IOMM-Lee is *NF2* wild-type but carries a BRAF V600E mutation and CDKN2A loss. Three patient derived cell lines (MN1, MN25, and MN26) harbor inactivating alterations of *NF2*.

Avutometinib (VS-6766) is a dual RAF/MEK inhibitor, with a unique ability to block both RAF and MEK kinase activity, thus inhibiting the MAPK signaling pathway more effectively than MEK inhibition alone. Defactinib (or VS-4718 preclinically) is a potent FAK inhibitor that disrupts tumor cell adhesion and survival signaling (Figure 1A). Previous studies have demonstrated enhanced efficacy by combining avutometinib and defactinib in various cancer models, including endometrial and ovarian cancer and melanoma.^15^^,^^16^^,^^19^^,^^20^ To the best of our knowledge, however, avutometinib or its combination with defactinib has never been tested for meningioma preclinically or clinically.

The primary goal of this study was to evaluate the therapeutic potential of defactinib and avutometinib as monotherapy and in combination in preclinical models of human meningioma. By leveraging both *in vitro* and *in vivo* approaches, we aimed to assess the agents’ synergistic interaction, anti-meningioma efficacy, and efficacy association with tumor genotypes. This study explored the potential for avutometinib and defactinib to address the unique challenges of meningioma treatment across genomically diverse models, including NF2-altered lines *in vitro* and MAPK-activated (BRAF V600E, NRAS Q61K) lines *in vivo*, and aims to establish a foundation for the clinical translation of dual-pathway inhibition in genomically defined meningioma subsets.

## Methods

### Cell Lines and Cell Cultures

Human meningioma cell line, IOMM-Lee (*NF2*-intact, RRID: CVCL_5779) was sourced from Dr. Randy Jensen at the University of Utah and American Type Culture Collection (ATCC). The CH157-MN cell line *(NF2*-deficient, RRID: CVCL_5723) was obtained from Dr. Wenya Bi at Brigham and Women’s Hospital. Ben-Men-1 cells were acquired from DSMZ (Braunschweig, Germany, RRID: CVCL_1959). All cell lines were maintained in DMEM supplemented with 10% fetal bovine serum (Cat# A3160402, Gibco) and 1X antibiotic-antimycotic solution (Cat# 15240062, Gibco). Cultures were incubated at 37°C in a 5% CO₂-humidified atmosphere and passaged upon reaching 80–90% confluence. Genetic profiling confirmed IOMM-Lee as *NF2*-wild type, while CH157-MN harbors deleterious *NF2* mutations and chromosome 22 loss, consistent with *NF2*-deficient meningiomas.^21^

To transduce firefly luciferase gene, IOMM-Lee cells were transduced with PLV-Firefly luciferase-mCherry (FmC) lentivirus, and CH157-MN cells were transduced with pGIPZ-Luc2-GFP lentivirus both in the presence of 8 μg/mL Polybrene (Sigma-Aldrich). Puromycin selection (1-2 μg/mL) started 72 hours post-transduction. Prior to implantation, IOMM-Lee transduced cells were sorted for mCherry by flow cytometry, and CH157-MN-Luc2-GFP cells were verified by luminescence signaling. Cell lines were authenticated via STR analysis and routinely tested for mycoplasma contamination using PCR-based assays and Lonza Mycoplasma Kit (Cat# LT07-418).

### Establishment of Patient-Derived Cell Cultures

The patient-derived meningioma models used for this study were MN1 *(NF2*-deficient*),* MN25 (*NF2*-deficient*)*, and M26 (*NF2*-deficient). These cells were established under protocols approved by Mayo Clinic Institutional Review Board and Massachusetts General Hospital (MGH) Institutional Review Board. Freshly resected surgical specimens were received from the operating room and enzymatically dissociated using gentleMACS Dissociator (Miltenyi Biotec) with 0.05% trypsin and DNase I, followed by washing with PBS, trituration, and filtration through a 100µM strainer to generate single-cell suspension that were placed directly into culture in the same conditions as IOMM-Lee and CH157-MN.

### Snapshot Profiling

Snapshot profiling was performed using a targeted next-generation sequencing assay based on anchored multiplex PCR to detect somatic single-nucleotide variants, small insertions/deletions, and gene-level copy number alterations. Genomic DNA was used to prepare sequencing libraries with a solid tumor–focused gene panel that were sequenced with paired-end reads on an Illumina platform. Sequence data were aligned to the hg19 human reference genome, and variants were identified and annotated using a validated bioinformatics pipeline^22^ (see Supplementary Methods).

### Cell Viability Assays

96-well plates were pre-coated with 25 µL of Corning® Matrigel® Growth Factor Reduced (GFR) Basement Membrane Matrix, LDEV-free (Catalogue number: 354230) per well and were incubated at 37°C with 5% CO₂ for 30 minutes to allow the Matrigel to solidify. Meningioma cells were then seeded into each well at a density of 1,000 cells per well. Following the overnight incubation, cells were treated with avutometinib and defactinib (both provided by Verastem Oncology), each prepared in DMEM media containing 2% Matrigel from 10 mM aliquots dissolved in DMSO. Serial dilutions of each drug, as well as their combination, were added to the cells. The plates were then incubated for 7 days. Drug response was assessed with cell viability measurement using the Promega CellTiter-Glo 3D Cell Viability Assay (Catalog number: G9681) and a Tecan Magellan plate reader. Synergy Combenefit normalized cell viability data, calculated as a percentage of vehicle-treated controls, were analyzed to compute synergy scores based on the Bliss independence model.^23^ Dose-response matrices were generated to evaluate synergistic, additive, or antagonistic effects across drug concentration combinations.

### Western Blot Analysis

IOMM-Lee and CH157-MN cells were cultured on Matrigel coating and treated for 4 hours with 3 µM defactinib and varying concentrations of avutometinib (0.3, 1, and 3 µM) in monotherapy and combination settings. Protein extraction was carried out as previously described.¹⁸ Proteins were separated by gel electrophoresis, transferred to PVDF membranes, and detected by immunoblotting using antibodies against phospho-ERK, ERK, phospho-FAK, FAK, NF2, and GAPDH (see Supplementary Methods).

### Western Blot Quantification

Densitometric analysis of Western blot bands was performed using ImageJ software (NIH). Protein expression levels were normalized to the corresponding loading control (β-actin), and relative intensities were reported as fold-change compared to control (see Supplementary Methods).

### In vivo Drug Preparation

Due to the poor pharmacokinetic properties of defactinib in mice, VS-4718, sourced from Verastem Oncology, was preferred for *in vivo* studies. VS-4718 is widely used as a surrogate for defactinib in mice.^8^^,^^19^^,^^24^^,^^25^ The vehicle solution for VS-4718 consisted of 0.5% carboxymethyl cellulose (CMC) (C5678, Sigma-Aldrich, St. Louis, MO) and 0.1% Tween 80 (P1754, Sigma-Aldrich, St. Louis, MO) dissolved in sterile water (B. Braun Medical, Irvine, CA, or equivalent). For *in vivo* administration, VS-4718 was dissolved in the vehicle solution to prepare a final concentration of 10 mg/mL. The resulting mixture was gently stirred continuously and sonicated in a bath-type sonicator for 5 to 10 minutes to ensure a uniform suspension. The prepared suspension could be stored at 2-8°C for up to one week.

The vehicle solution for avutometinib was composed of 5% dimethyl sulfoxide (DMSO) and 10% hydroxypropyl-β-cyclodextrin (HPCD) in sterile water. For drug preparation, avutometinib was dissolved in the vehicle solution to prepare a final concentration of 0.06 mg/mL. The mixture was briefly vortexed or sonicated using a bath-type sonicator to create a uniform solution. This preparation was stored at 2-8°C for up to 1week.

### Animal Studies

All animal studies were conducted in strict compliance with the guidelines and regulations set forth by the Institutional Animal Care and Use Committee (IACUC) at Massachusetts General Hospital (MGH) and Mayo Clinic Rochester (MCR), which approved the experimental protocols. Six to 8-week-old female athymic nude (nu/nu) mice were sourced from Charles River Laboratories (RRID: IMSR_CRL:490) and housed in Biosafety level 2 animal facility. The mice were maintained on a 12-hour light-dark cycle with unrestricted access to food and water.

### In vivo Efficacy Studies in Flank Meningioma Models

Mice were subcutaneously implanted with 1 × 10^6^ CH157-MN or IOMM-Lee meningioma cells mixed with Matrigel at 1:1 (total 100 µL per mouse) in the right flank, and tumors were allowed to establish before treatment initiation. Tumor-bearing mice were randomized into 4 groups: vehicle, VS-4718 monotherapy, avutometinib monotherapy, and combination (*n* = 9 per group for CH157-MN xenografts and *n* = 7 per group for IOMM-Lee xenografts). Daily oral administration of VS-4718 (50 mg/kg or vehicle twice daily via oral gavage), avutometinib (0.3 mg/kg, or vehicle once daily via oral gavage), and their combination began 7 days post-implantation and continued for 4 weeks. Weekend dosing was limited to morning administrations. Tumor diameters were measured every other day by digital calipers. Tumor volume was calculated using the modified ellipsoid formula V = (L × W^2^)/ 2 (for IOMM-Lee model) or (L × W × H)/ 2 (for CH157-MN model), where L, W, and H represent the longest, intermediate, and shortest tumor dimensions, respectively. Animals were monitored closely for signs of distress, weight loss, or tumor ulceration. Animals were euthanized if they developed tumors that were greater than 20 mm in diameter or tumor ulceration covered more than 50% of the tumor surface area, if deep tissue within the tumor site became exposed, or if signs of infection, such as redness, swelling, or discharge, were observed. Additionally, necrosis, indicated by the presence of black tissue, or tumor growth that restricted mobility or access to food and water were considered criteria for euthanasia.

### Intracranial Implantation of Meningioma Cells in Mice

Mice were given an analgesic with children’s ibuprofen at 0.2 mg/mL in drinking water or meloxicam (10 mg/kg subcutaneously) and anesthetized intraperitoneally with ketamine/xylazine (90 mg/kg and 10 mg/kg, respectively). They were secured in a stereotactic frame. A small incision was made on the scalp to expose the skull, and a burr hole was drilled at the target coordinates relative to bregma: AP = 0.0 mm, ML= −2.5 mm, and DV = 1.0 mm. A cell dose of 1 × 10⁵ meningioma cells was prepared in a 1-2 µL of HBSS and loaded into a Hamilton syringe. The cell suspension was carefully injected at a rate of approximately 1-2 µL/min. After the injection, the burr hole was sealed with bone wax. The incision was closed with either wound clips or 5-0 vicryl sutures. Following surgery, the mouse was placed on a heated blanket until fully recovered from anesthesia. Post-operative analgesics were continued for 72 hours postoperatively.

### In vivo Pharmacokinetics and Pharmacodynamics Study

Pharmacokinetics (PK) and pharmacodynamics (PD) of VS-4718 and avutometinib were evaluated using mice bearing IOMM-Lee-FmC intracranial tumors as described in Supplementary Methods.

### In vivo Efficacy Studies in Intracranial Meningioma Models

Mice with established intracranial tumors were given daily oral administration of VS-4718 (50 mg/kg; twice daily), avutometinib (0.3 mg/kg; once daily), or their combination, starting five days after tumor implantation. Treatments continued for 4 weeks, with weekend dosing restricted to mornings. Animals were weighed 3 times per week to ensure animal welfare and monitor overall health during the study. Supportive care measures were implemented to mitigate drug toxicity and address moderate weight loss, including dietary supplementation with diet gel provided for 3 weeks post-implantation and while animals received treatment. Additional supportive care was given to animals showing signs of weight loss, including subcutaneous injections of 800 µL saline and oral administration of 100 µL NutriCal if body weight dropped to 92% of the maximum recorded weight, or twice-daily supportive care with the same doses if body weight fell below 20 g. Dosing was discontinued if weight loss was accompanied by a reduction of 0.5 g within 24 hours or if body weight fell to 90% of the maximum. Humane euthanasia was performed if animals exhibited a 20% reduction in body weight from the initial body weight at the start of treatment or developed significant neurological deficits.

### Bioluminescent Imaging

Bioluminescent imaging (BLI) was performed 2-7 days after tumor implantation and at 5-7-day intervals during the dosing period. For IOMM-Lee-FmC mice, luciferin (150 mg/kg, i.p.) was used as the substrate; Cycluc1 (10 mg/kg, i.p.) was used for CH157-MN-GFP-Luc2 mice. BLI signals were quantified in units of radiance (photons/sec/cm²/sr). ROI analysis was performed as described in Supplementary Methods. CH157-MN-GFP-Luc2 images were analyzed as previously described.^19^

### Statistical Analyses

Data were presented as mean ± SEM. Dose-response curves were generated using nonlinear regression curve fit function in GraphPad Prism. For *in vitro* assays and BLI radiance, comparisons between 2 groups were made using the two-sample test (rank-sum). Differences between more than 2 groups were determined using One-Way ANOVA with Tukey’s multiple comparison tests. Tumor growth inhibition was assessed using tumor volume measurements. Comparisons across groups were made using analysis of variance (Kruskal-Wallis) and the two-sample t-test (ranks-sum) as appropriate. Appropriate transforms were applied to fit model distributional assumptions. Tumor volume and weight at select time points were compared across groups using analysis of covariance, adjusting for baseline tumor volume or weight as appropriate. Within-group comparisons for tumor volume and weight for specific time points (eg weight on day 7 versus weight on day 1) were made using the paired t-test (signed rank) as appropriate. For time-to-event time points, cumulative survival probabilities were estimated using the Kaplan-Meier method; the log rank test was used to make comparisons across groups. In all cases, *P*-values < .05 were considered statistically significant.

## Results

### In vitro Effects of Defactinib and Avutometinib on Meningioma Cell Viability and Signaling

To access the therapeutic efficacy of defactinib and avutometinib in meningioma cell lines, we evaluated effects on cell viability in two established meningioma cell lines, IOMM-Lee (*NF2* wild-type, BRAF V600E, and *CDKN2A* homozygous deletion) and CH157-MN (*NF2* mutant and *NRAS* mutant), using *in vitro* assays. CH157-MN cells were derived from a recurrent meningioma (WHO Grade unknown), whereas IOMM-Lee were established from an anaplastic meningioma (WHO Grade 3) (Figure 1B). Both cell lines harbor *TERT* promoter mutations, which, according to the current WHO 2021 criteria, are sufficient to classify a meningioma as grade 3 regardless of histology.^26^ These cell lines exhibit aggressive growth characteristics and have been extensively utilized for studying meningioma progression and therapeutic efficacy. The cytotoxic effects of defactinib and avutometinib on meningioma cells were assessed in the presence of Matrigel plate surface coating since FAK signaling is dependent on cues provided by extracellular matrix.^27^ Both defactinib and avutometinib monotherapy decreased cell viability in IOMM-Lee and CH157-MN cells in a dose-dependent manner (Figure 2A). Avutometinib showed greater activity in IOMM-Lee cells than in CH157-MN cells, indicating that IOMM-Lee was more sensitive to avutometinib than CH157-MN (Figure 2A).

**Figure 2.**
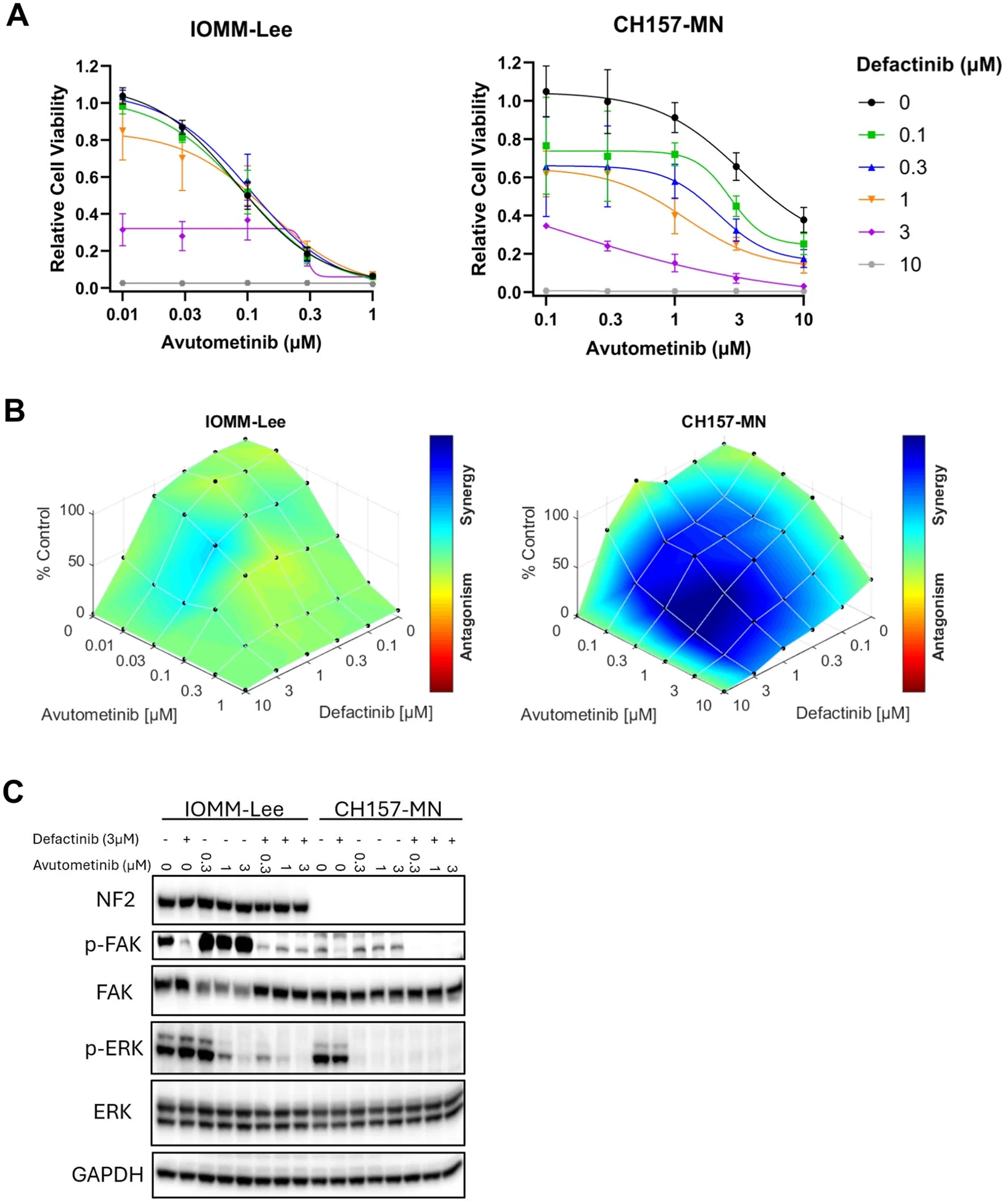
Effects of defactinib and avutometinib on meningioma cell lines *in vitro*. (A) Effects of defactinib and avutometinib on cell viability in IOMM-Lee (left) and CH157-MN (right). Cells were treated with increasing concentrations of defactinib or avutometinib alone or in combination for 7-days on matrigel coated plates. Data are presented as mean ± SEM. (B) Bliss synergy maps of and IOMM-Lee (left) CH157-MN (right) cells treated with defactinib and avutometinib. (C) Western blot analysis of pathway inhibition in IOMM-Lee and CH157-MN meningioma cells treated with defactinib and avutometinib for 4 hours in the presence of matrigel.

Given the differential responses to monotherapy, we next explored potential synergistic effects by analyzing combined defactinib and avutometinib treatment in IOMM-Lee and CH157-MN cells using the BLISS synergy model. Drug interactions between defactinib and avutometinib were mostly additive in the IOMM-Lee cells (Figure 2B). On the other hand, the combination showed a potent synergistic effect in the CH157-MN cells across a wide range of drug concentrations (Figure 2B).

To understand the molecular mechanisms underlying these effects, we examined the cell signaling impact of defactinib and avutometinib by analyzing p-FAK and p-ERK expression, the key markers of FAK and RAF/MEK signaling, via Western blot in both cell lines. Expression p-FAK was higher in IOMM-Lee compared with CH157-MN cells, and p-FAK was inhibited by defactinib (3 µM) in both cell lines (Figure 2C). Treatment of CH157-MN cells with avutometinib completely inhibited p-ERK across all tested concentrations (0.3-3 µM). In IOMM-Lee cells, however, higher concentrations of avutometinib (≥ 1µM) were required to achieve a similar level of p-ERK suppression observed in CH157-MN cells. Avutometinib induced an upregulation of p-FAK across the concentrations tested (0.3-3 µM) in IOMM-Lee cells, suggesting compensatory activation of FAK signaling in response to the blockade of RAF/MEK signaling as previously reported.^28^ Combination of defactinib and avutometinib effectively abrogated this signaling crosstalk and achieved inhibition of both p-FAK and p-ERK in both IOMM-Lee and CH157-MN cells.

To assess whether the observed sensitivity to avutometinib and defactinib in IOMM-Lee and CH157-MN was associated with the presence of MAPK pathway mutations, an additional established cell line, Ben-Men-1, was evaluated as it has *NF2* loss and lacks MAPK pathway alteration (Figure 1B). Ben-Men-1 exhibited resistance to avutometinib, whereas defactinib had a greater effect. When the drugs were combined, interaction was additive or antagonistic at higher avutometinib doses (Supplementary Figure 1B).

### Patient-Derived NF2-Mutant Meningioma Cells Responded to Defactinib but Were Resistant to Avutometinib

To apply these drugs to more clinically applicable models, we tested three cell models derived from patients with high-grade meningioma: MN1, MN25, and MN26 (Figure 1B; Supplementary Figure 2). These meningioma cell lines underwent targeted genetic profiling, which found that all 3 models harbored *NF2* mutations (Figure 1B). Additionally, MN1 had *TP53* and *TERT* promoter mutations. MN26 was found to have a *KRAS* variant of unknown significance (Figure 1B).

These cell lines were treated with defactinib and avutometinib with the same Matrigel environment used for the established cell lines for cell viability assays. Similar to the established cell lines, the patient-derived cell lines responded to defactinib monotherapy effectively (Figure 3A-C). However, similar to the response observed in Ben-Men-1 cells, all the patient-derived cell lines were resistant to avutometinib, showing no appreciable response to the concentration range tested (0.001 to 10 µM) (Figure 3A-C). Rather, in MN25 and MN26, avutometinib paradoxically increased cell viability. Overall, the *NF2*-mutant meningioma models lacking MAPK alterations were insensitive to avutometinib but showed greater sensitivity to defactinib than the IOMM-Lee and CH157-MN cell lines.

**Figure 3.**
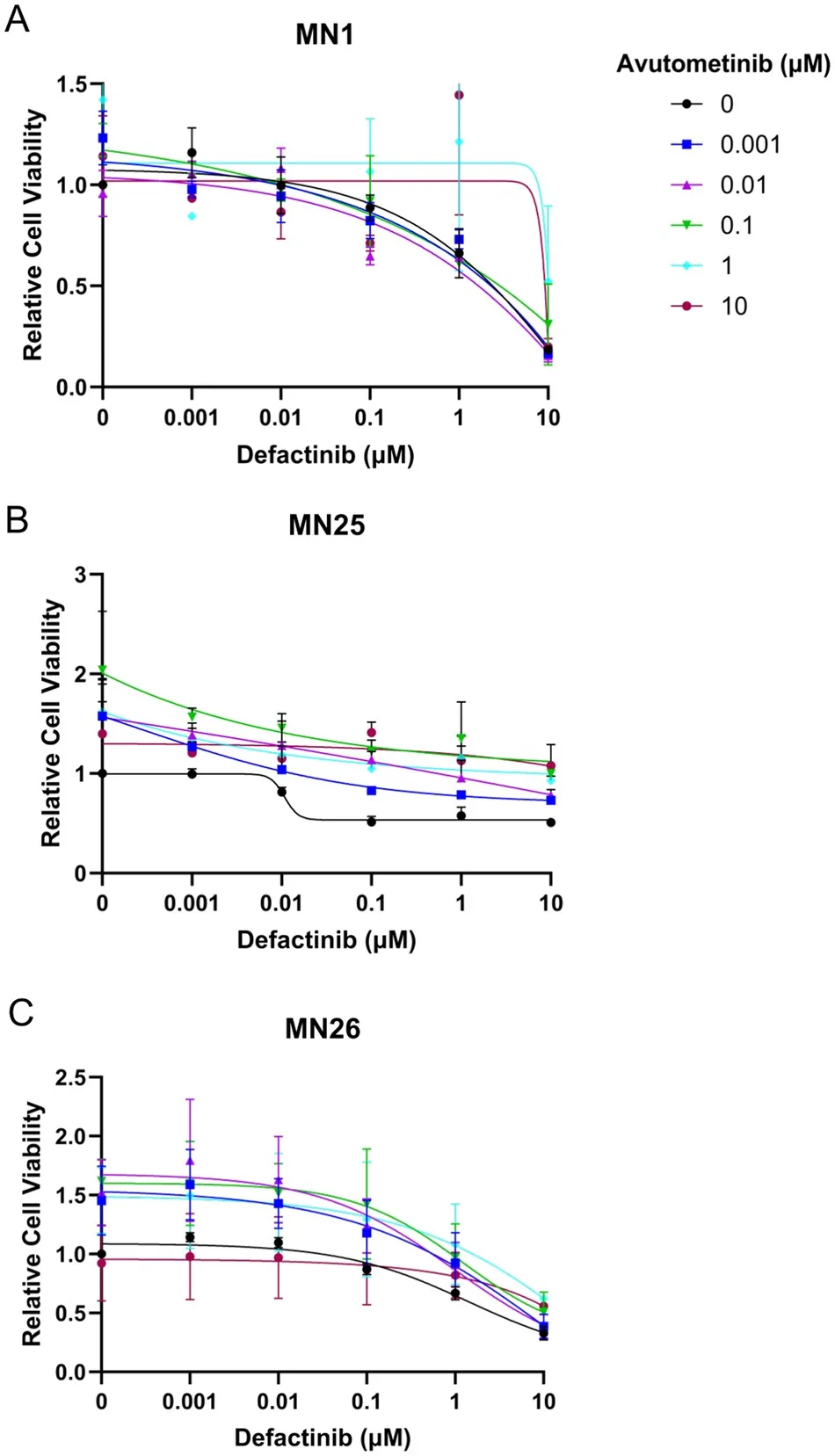
Effects of defactinib and avutometinib in patient-derived meningioma cells. (A) MN1, (B) MN25, and (C) MN26 cells were treated with increasing concentrations of defactinib or avutometinib alone or in combination for 7-days on matrigel coated plates and cell viability evaluated using Cell-Titer Glo. Data are presented as mean ± SEM.

### Combination Therapy with VS-4718 and Avutometinib Reduces Tumor Growth and Extends Survival in Flank Models of Meningioma Xenografts

We next determined whether the activity and interaction of defactinib and avutometinib found *in vitro* with CH157-MN and IOMM-Lee could be translated into *in vivo* efficacy. These meningioma cells were first subcutaneously implanted into nude mice to generate flank tumor models. VS-4718 was selected as a substitute for defactinib due to its more favorable pharmacokinetics in preclinical mouses studies.^19^^,^^24^ After tumor establishment, mice were randomized into 4 treatment groups: vehicle control, VS-4718 (50 mg/kg twice a day) monotherapy, avutometinib (0.3 mg/kg once a day) monotherapy, and combination therapy (50 mg/kg VS-4718 twice a day + 0.3 mg/kg avutometinib once a day) (Figure 4A). Animals were administered daily treatments for 4 weeks. Tumor volumes were measured throughout the study and monitored until the defined experimental endpoints.

**Figure 4.**
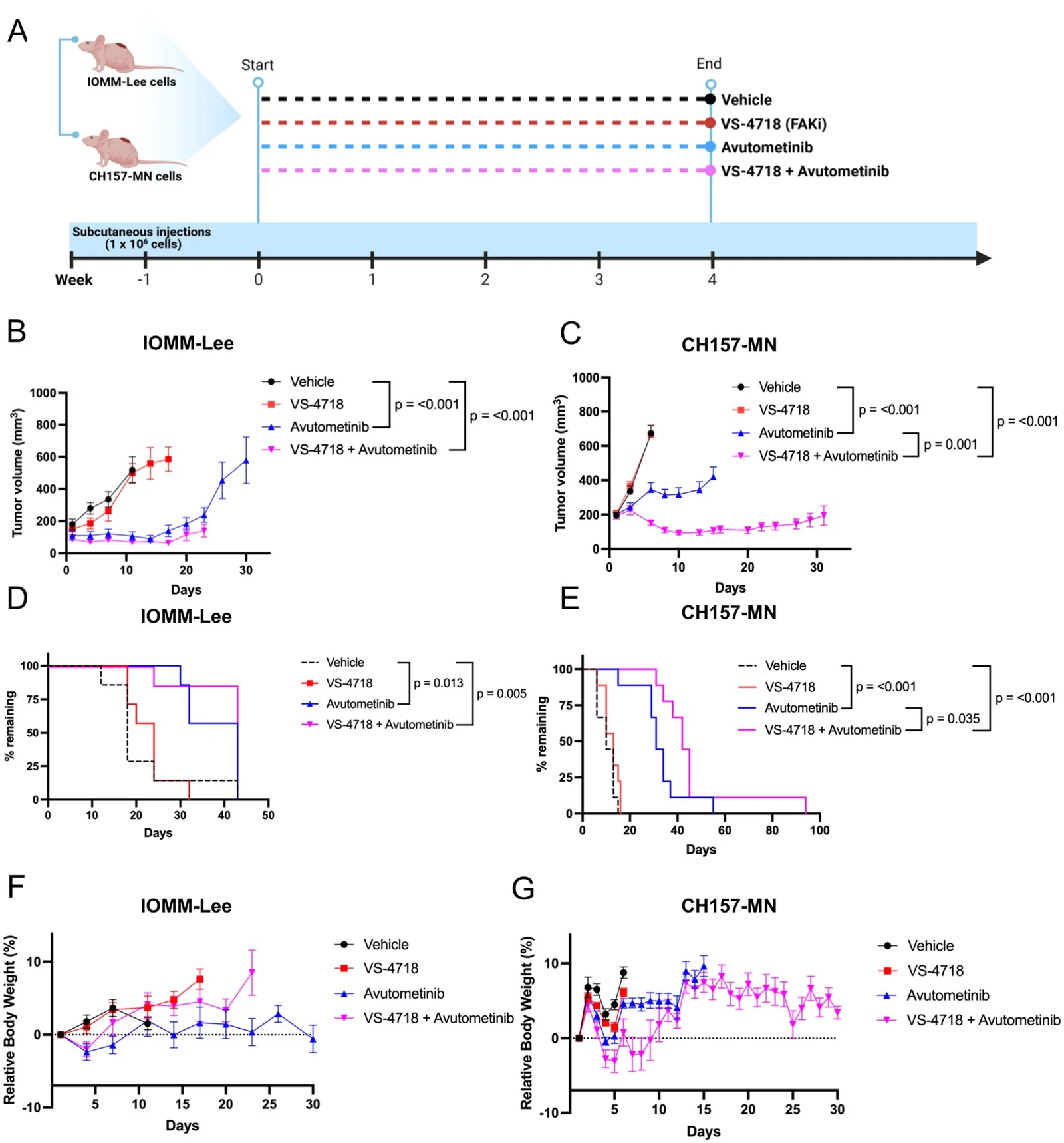
Efficacy of VS-4718 and avutometinib in IOMM-Lee and CH157-MN subcutaneous tumor models. (A) Schematic representation of the subcutaneous flank implantation experiment. IOMM-Lee (*NF2* wild-type, BRAF p.V600E) and CH157-MN (*NF2* mutant, NRAS p.Q61K) meningioma cells (1 × 10⁶ cells) were injected subcutaneously into the flanks of female nu/nu mice. Following tumor establishment (5 days), mice were randomized into 4 treatment groups: Vehicle (control), VS-4718 (FAK inhibitor), avutometinib (RAF/MEK inhibitor), and combination VS-4718 + avutometinib. Treatments were administered daily for 4 weeks. (B, C) Tumor volume measurements over time in IOMM-Lee (B) and CH157-MN (C) models. Tumor growth was tracked in each treatment group. *P*-values reflect between-group comparisons at day 11 (4B) and day 6 (4C), adjusting for baseline volume. (D, E) Kaplan-Meier survival curves of IOMM-Lee (D) and CH157-MN (E) tumor-bearing mice. Survival was assessed in each treatment group over time. (F, G) Body weight changes over time in mice of each treatment group with IOMM-Lee (F) and CH157-MN (G) subcutaneous meningioma. Data are presented as mean ± SEM.

In IOMM-Lee xenografts, avutometinib monotherapy significantly reduced flank tumor growth compared to vehicle (*P* < .001), whereas VS-4718 monotherapy showed no significant effect (*P* = .68) (Figure 4B). Combination therapy also significantly reduced tumor growth (*P* < .001) (Figure 4B), with no statistically significant difference between avutometinib monotherapy and combination treatment (*P* = .95). Similarly, in CH157-MN xenografts, vehicle-treated mice exhibited rapid tumor progression, with average tumor size reaching approximately 700 mm³ by day 6 (Figure 4C). Avutometinib monotherapy significantly slowed tumor growth (*P* < .001), while combination therapy halted tumor growth over the 4-week treatment period and significantly enhanced tumor inhibition over avutometinib alone (*P* = .001; Figure 4C). VS-4718 monotherapy had no significant impact on tumor growth in the CH157-MN model (*P* = .72).

In IOMM-Lee xenografts, Kaplan-Meier analysis of overall survival demonstrated that avutometinib significantly extended survival relative to vehicle both as monotherapy and in combination with VS-4718 (*P* = .013 and .005, respectively), but VS-4718 monotherapy did not improve survival (*P* = .66; overall *P* = .0004; Figure 4D). In CH157-MN xenografts, avutometinib monotherapy significantly extended survival relative to vehicle (*P* < .001), while VS-4718 monotherapy showed no significant survival benefit (*P* = .33; overall *P* < .001; Figure 4E). The combination of VS-4718 and avutometinib resulted in the longest survival in this model, with a significant improvement over avutometinib monotherapy (*P* = .035; Figure 4E).

In IOMM-Lee tumor-bearing mice, body weight remained stable in the vehicle and VS-4718 groups throughout the study period (Figure 4F). Mice treated with avutometinib and the combination therapy exhibited a mild weight loss, reaching approximately 2%-3% below baseline around days 5-6, followed by rapid recovery. Similarly, in CH157-MN tumor-bearing mice, the combination group showed a peak weight reduction of ∼3% by days 6-7 (Figure 4G). The avutometinib monotherapy group exhibited a milder, transient decrease, while weights in the vehicle and VS-4718 groups remained stable.

Overall, avutometinib monotherapy significantly reduced tumor growth in both IOMM-Lee and CH157-MN meningioma flank xenografts, while VS-4718 alone was ineffective. The combination of avutometinib and VS-4718 exhibited enhanced efficacy compared with avutometinib alone in the CH157-MN model.

### Pharmacokinetics and Pharmacodynamics of VS-4718 and Avutometinib in Intracranial Meningioma Xenografts

We next conducted a pharmacokinetics study of VS-4718 and avutometinib in mice bearing intracranial IOMM-Lee xenografts. Following three days of treatment, the mice were euthanized three hours after the final dose to collect blood and tumor samples (Figure 5A). Plasma concentration of avutometinib was 407 ng/g in the monotherapy group and 387 ng/g in the combination group, while the corresponding tumor concentrations were 119 ng/g and 143 ng/g, respectively. These values yielded Kp ratios (tumor-to-plasma) of 0.26 in monotherapy and 0.37 in the combination group (Figure 5B). For VS-4718, plasma concentrations were 1,059 ng/g (monotherapy) and 1,132 ng/g (combo), with tumor concentrations of 488 ng/g and 562 ng/g, corresponding to Kp values of 0.46 and 0.50, respectively (Figure 5C). Comparable Kp values observed in the monotherapy and combination therapy groups for both agents suggested a lack of detrimental drug interaction between VS-4718 and avutometinib that may have impacted tumor exposure to the agents.

**Figure 5.**
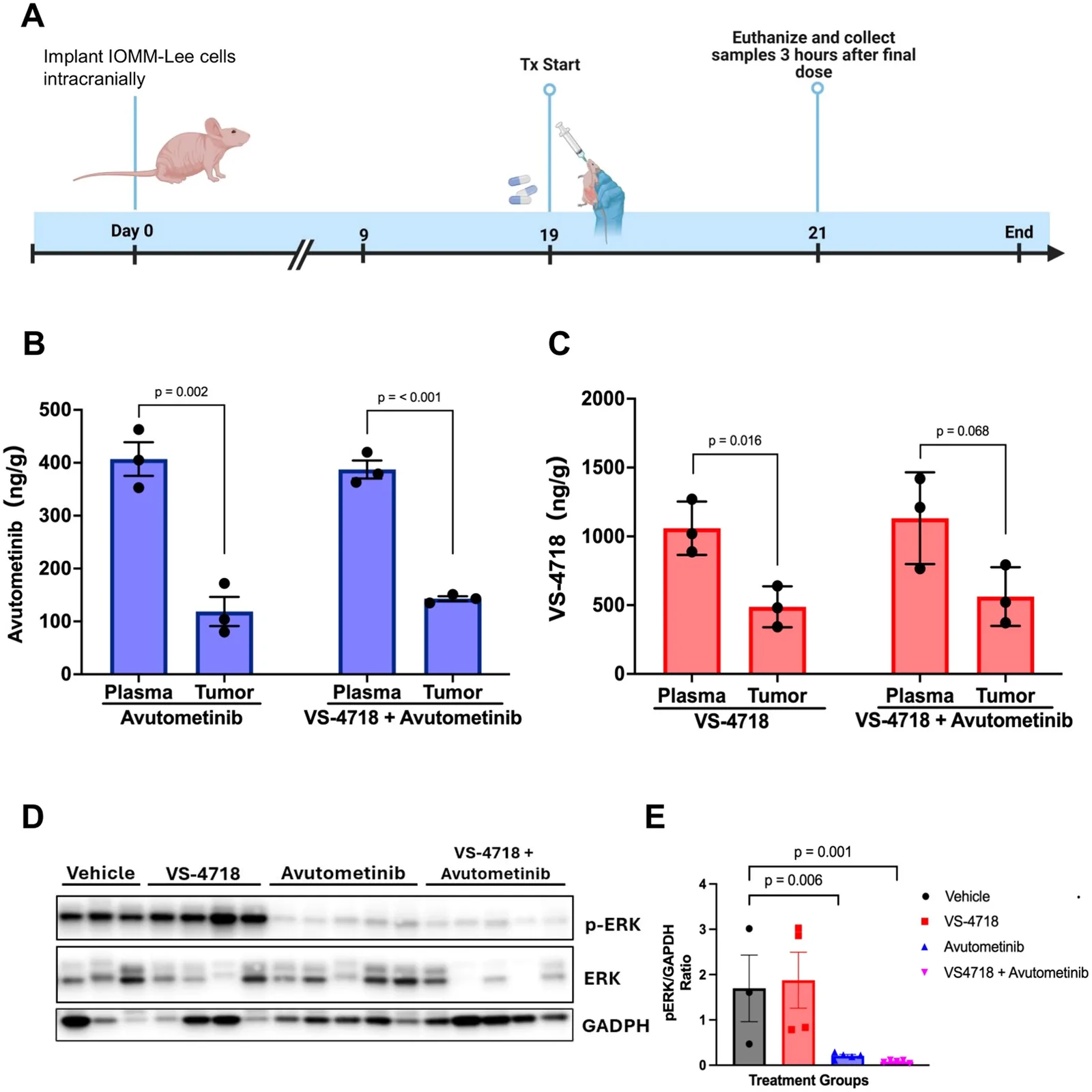
Pharmacokinetic (PK) and pharmacodynamic (PD) analyses of avutometinib and VS-4718 in plasma and intracranial IOMM-Lee tumors. (A) Schematic illustration of the experimental design. (B and C) Concentrations of avutometinib (B) and VS-4718 (C) in plasma and intracranial tumor tissue. Data are presented as mean ± SEM. (D) Western blot of p-ERK and ERK in intracranial tumor tissues after treatment. GAPDH, loading control. (E) Quantification of pERK/GAPDH ratios in tumor samples (from D). Each data point represents an individual sample, and error bars indicate SEM. Statistical comparisons between treatment groups were performed using one-way ANOVA.

Having confirmed that VS-4718 and avutometinib achieve adequate tumor concentrations, we proceeded to evaluate their pharmacodynamic effects. We investigated the effects of VS-4718 and avutometinib, individually and in combination, on the phosphorylation of ERK using Western blots of intracranial samples and image quantitation. In the vehicle group, levels of p-ERK showed variability, with p-ERK/GAPDH ranging from 0.47 to 3.01, indicating heterogeneous baseline activation across the intracranial samples (Figure 5D, E). Treatment with VS-4718 alone did not alter p-ERK/GAPDH levels (Figure 5D, E). In contrast, avutometinib alone effectively suppressed p-ERK (p-ERK/GAPDH: 0.11-0.29 with *P* = .006) (Figure 5D, E). The combination of VS-4718 and avutometinib consistently and potently suppressed p-ERK/GAPDH lowering it to 0.03-0.11 (Figure 5D, E). These findings highlight the potent on-target inhibition of MAPK/ERK by avutometinib, with enhanced effects from the combination treatment in meningioma.

### Combination Treatment of FAK and RAF/MEK Inhibitors in Xenograft Models of Intracranial Meningioma

We next evaluated the effectiveness of avutometinib and VS-4718 in intracranial meningioma xenografts. Luciferase-tagged IOMM-Lee or CH157-MN cells were injected intracranially to establish orthotopic tumors in nude mice. As in flank studies, the mice were divided into 4 treatment groups: vehicle (control), VS-4718 monotherapy, avutometinib monotherapy, and the combination therapy receiving both VS-4718 and avutometinib (Figure 6A). Tumor burden was serially assessed by BLI before treatment start, midway through, and upon completion of total 4 weeks of daily treatments.

**Figure 6.**
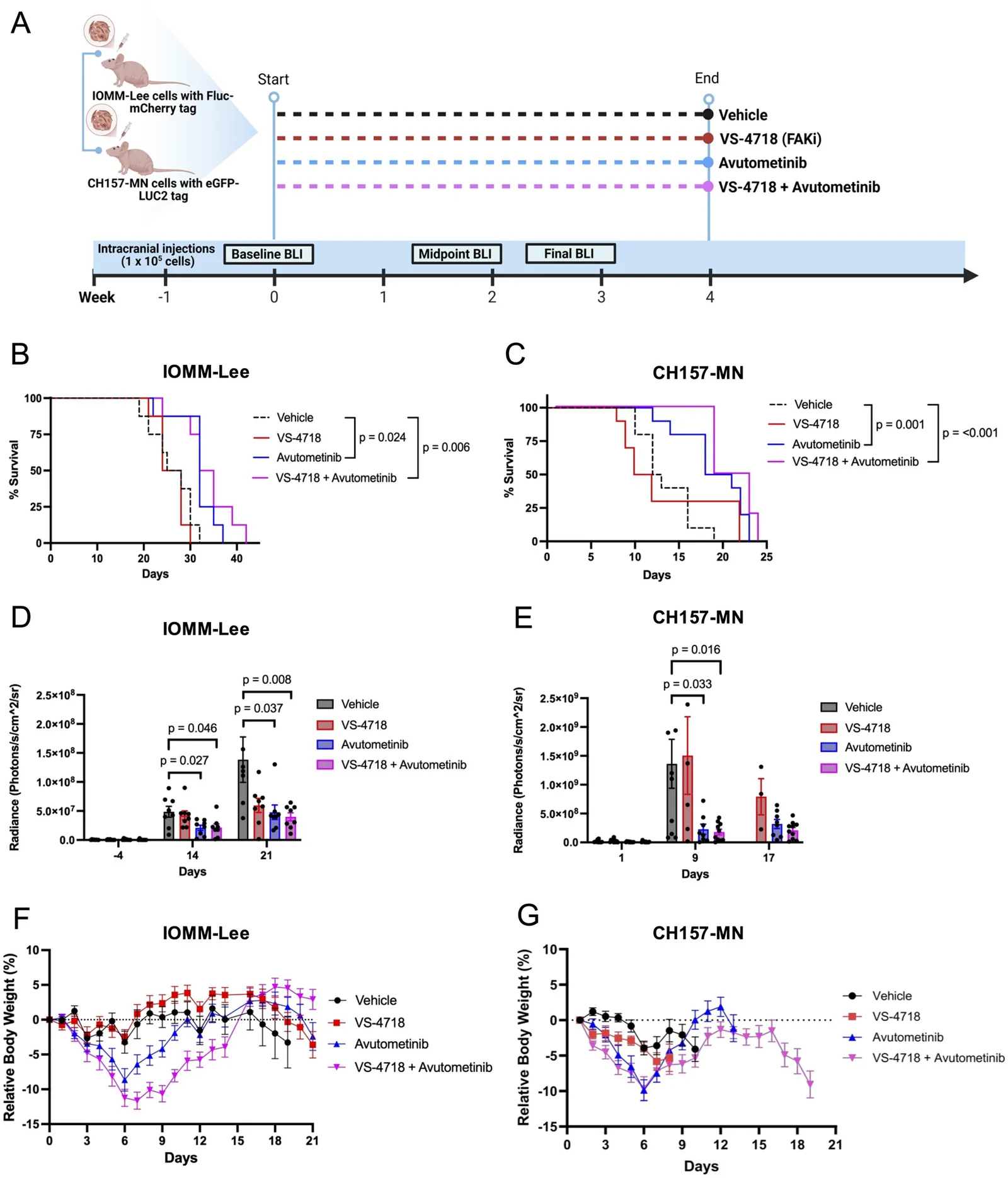
Therapeutic efficacy of VS-4718 and avutometinib in intracranial meningioma models. (A) Schematic of the intracranial tumor model and treatment timeline. IOMM-Lee (*NF2* wild-type, BRAF p.V600E) and CH157-MN (*NF2* mutant, NRAS p.Q61K) meningioma cells expressing firefly luciferase were injected intracranially into nude mice (1 × 10⁵ cells). Treatment began 7 days post-implantation and was administered daily for 4 weeks. Bioluminescence imaging (BLI) was performed at baseline, midpoint, and endpoint to monitor treatment effects and tumor progression. Mice were randomized into 4 treatment groups: Vehicle, VS-4718 (FAK inhibitor), avutometinib, and combination VS-4718 + avutometinib. (B, C) Kaplan-Meier survival curves of IOMM-Lee (B) and CH157-MN (C) tumor-bearing mice. Survival was monitored over time for each treatment group. (D, E) BLI signal quantification in IOMM-Lee (D) and CH157-MN (E) models. BLI signal (radiance, photons/sec/cm²/sr) was measured to assess tumor burden. (F, G) Relative body weight changes over time in IOMM-Lee (F) and CH157-MN (G) models. Body weight (%) was recorded throughout the study to assess treatment tolerability. Data are presented as mean ± SEM.

In the IOMM-Lee model, avutometinib alone extended median survival to 32 days from 27 days in the vehicle group (*P* = .024) (Figure 6B). The combination of avutometinib and VS-4718 similarly enhanced survival, with the median survival time of 33.5 days (*P* = .006 compared with vehicle). In contrast, VS-4718 alone was not significantly (*P* = .21) different from the vehicle (Figure 6B). In the CH157-MN model, treatment with avutometinib significantly extended median survival from approximately 12.5 days in the vehicle group to 19 days (*P* = 0.001) (Figure 6C). The combination therapy also improved survival over the vehicle, with a median survival of 20 days (*P* < .001), which was not different from avutometinib monotherapy (Figure 6C). VS-4718 monotherapy showed no significant difference (*P* = 0.81) from the vehicle (Figure 6C).

Intracranial tumor analysis using BLI was consistent with these survival outcomes. In the IOMM-Lee model, regimens involving avutometinib (monotherapy and combination with VS-4718) significantly inhibited tumor growth, with radiance reductions observed on days 14 (*P* = .046 and *P* = .027, respectively) and 21 (*P* = .008 and *P* = .037, respectively) compared with vehicle (Figure 6D, Supplementary Figure 3). Similarly, in the CH157-MN model, both avutometinib monotherapy and combination therapy significantly suppressed tumor growth, as evidenced by reduced radiance on day 9 (*P* = .033 and .016, respectively), compared to the vehicle (Figure 6E, Supplementary Figure 4). Tumor burden was comparable between VS-4718 alone and the vehicle, except decreased radiance being observed in the VS-4718 group on day 21 (*P* = .049) in the IOMM-Lee model (Figure 6D, E).

Body weight monitoring showed a transient weight loss of approximately 10% around day 6-7 in the combination group (avutometinib and VS-4718) affecting 6/7 mice in IOMM-Lee model and 4/10 mice in the CH157-MN model, with milder effects observed in the avutometinib monotherapy group (3/7 mice in IOMM-Lee and 5/10 mice in CH157-MN) (Figure 6F, G). To ensure animal welfare, dosing was temporarily held per protocol if an animal’s weight loss exceeded 10% and resumed upon recovery.

Together, avutometinib demonstrated significant efficacy in suppressing tumor growth and extending survival in both intracranial meningioma xenografts, with notable effects in the *NF2*-mutant CH157-MN model. However, the combination therapy with VS-4718 was associated with tolerability challenges.

## Discussion

A significant subset of meningiomas, including high-grade and recurrent tumors, display aggressive clinical behavior and limited responsiveness to existing treatments. These therapeutic limitations highlight an urgent need for new, mechanism-driven approaches as well as incorporating new emerging molecular classifications in diagnosing meningiomas that can address the biologic heterogeneity and therapeutic resistance commonly observed in patients with meningioma.

Our study evaluates a dual-inhibition strategy targeting two interconnected survival pathways commonly implicated in tumor growth and resistance: the MAPK/ERK pathway, inhibited by avutometinib, and the FAK pathway, inhibited by defactinib/VS-4718. The rationale for this approach stems from prior evidence that MAPK inhibition can lead to compensatory FAK activation, allowing cells to evade treatment.^16^ Although this adaptive resistance has been most thoroughly characterized in RAS/RAF-driven cancers, similar MAPK–FAK crosstalk has been observed in other tumors, including meningiomas, particularly in the context of *NF2* loss, where merlin inactivation leads to constitutive FAK pathway signaling.^8^^,^^10^

Preclinical studies in models of ovarian, uveal melanoma, and KRAS mutant cancers have shown that co-inhibition of MAPK and FAK signaling produces synergistic anti-tumor effects by enhancing apoptosis and limiting compensatory resistance. Notably, the combination of avutometinib and defactinib has demonstrated encouraging clinical activity in the FRAME Phase 1/2 trial for low-grade serous ovarian cancer, achieving a 42.3% objective response rate and extended progression-free survival (NCT03875820).^29^ Additionally, a phase II trial using the FAK inhibitor GSK2256098 in patients with *NF2-*mutant meningioma demonstrated promising 6-month progression-free survival rates (33%-83%),^3^ supporting FAK as a viable therapeutic target in these tumors. Building on this foundation, our study is the first to systematically assess this dual-targeting approach in preclinical models of human meningioma.

### In Vitro Evidence of Synergy and Heterogeneity

Our Western blot analysis validated the mechanistic basis for dual inhibition, revealing reciprocal pathway activation; MAPK inhibition with avutometinib increased pFAK levels, while FAK inhibition with defactinib appeared to have upregulated pERK in IOMM-Lee cells. Both FAK inhibition and MAPK inhibition have been shown to induce apoptosis in monotreatment and combination.^30^^,^^31^ These findings underscore the rationale for co-targeting these pathways to disrupt compensatory survival mechanisms. However, the degree of synergy mediated by defactinib and avutometinib varied between cell lines, reflecting possible differences in pathway dependence. Potent synergy was noted in CH157-MN cells *in vitro*. The greater sensitivity to the combination in CH157-MN may reflect its reliance on dysregulated MAPK and FAK pathways for survival.

In contrast, the three *NF2*-mutant patient-derived cultures (MN1, MN25, and MN26) exhibited resistance to avutometinib monotherapy. These distinct responses *in vitro* between the established cell lines and the patient-derived cell lines might be explained by the genetic differences that could underlie the variable sensitivity to avutometinib and defactinib. CH157-MN and IOMM-Lee harbor MAPK mutations, whereas the patient-derived lines were primarily driven by *NF2* loss without alterations in the MAPK pathway. Although MN26 harbors a rare *KRAS* variant of uncertain significance (p. Val186Leu), which lacks reported functional evidence of pathway activation.^32^ Although NF2 loss may lead to indirect upregulation of MAPK signaling through merlin’s upstream regulatory role, without a constitutively activating mutation in the MAPK pathway, dependence appears insufficient to confer sensitivity to avutometinib monotherapy.

To further explain how the dysregulated MAPK signaling creates sensitivity and the observed synergy, an additional established cell line, Ben-Men-1, was tested, which lacks MAPK mutations and harbors *NF2* loss like the patient-derived cells. Ben-Men-1 demonstrated minimal response to avutometinib monotherapy and no synergistic interaction. Ben-Men-1, however, was shown to be sensitive to the inhibition of mTORC1, a downstream effector in the MAPK signaling pathway.^33^ Collectively, our findings indicate that the *in vitro* synergy seen in CH157-MN and IOMM-Lee likely relies on hyperactivity of the MAPK signaling, which may not be representative of most *NF2*-driven meningiomas.

### Flank and Intracranial *In Vivo* Models Highlight Activity and Limitations

Defactinib, while clinically used in humans, demonstrates poor pharmacokinetic properties in mice, limiting its utility in preclinical *in vivo* studies. VS-4718, a structurally related FAK inhibitor, and defactainib show similar responses across cancer cell lines *in vitro.*^34^ VS-4718 has been widely used as a murine surrogate for defactinib across multiple preclinical cancer models and was therefore selected for all *in vivo* experiments in this study.^8^^,^^19^^,^^24^^,^^25^

While defactinib reduced the growth of IOMM-Lee cells *in vitro*, FAK inhibition on its own showed limited efficacy *in vivo*. The difference between *in vitro* and *in vivo* results has been observed with FAK inhibitors and may reflect the complex tumor microenvironment.^24^

*In vivo* studies showed that avutometinib was consistently effective in heterotopic and orthotopic models of *NF2*-mutant CH157-MN and *NF2*-wildtype IOMM-Lee cells. In the flank CH157-MN model, the combination of avutometinib and VS-4718 achieved greater tumor suppression than avutometinib monotherapy. This translated into substantial survival benefit, with median survival more than double that of vehicle or single-agent treatment. However, in the intracranial model, the survival benefit was less pronounced, and combination treatment did not significantly improve survival compared to avutometinib monotherapy. These findings suggest that while dual pathway inhibition may offer enhanced efficacy in certain contexts, the synergistic benefit observed *in vitro* and in the flank models may not extend to intracranial benefit. The reduced efficacy in orthotopic models may reflect multiple factors, including differences in the tumor microenvironment, potential regional limitations in drug distribution within the intracranial tumor despite overall favorable pharmacokinetics, or poorer tolerability of the combination in mice with orthotopic tumors.

Both avutometinib and VS-4718 show favorable pharmacokinetics in orthotopic meningioma models. Relatively high tumor-to-plasma Kp values were observed for both avutometinib and VS-4718, consistent with drug penetration into tumor, and no detrimental drug-drug interaction. Accordingly, pharmacodynamic analysis confirmed on-target inhibition of p-ERK by avutometinib. However, combination therapy was associated with tolerability concerns, particularly in the intracranial models. Significant weight loss was observed, leading to missed doses or reduced dose intensity, which may have contributed to attenuated survival benefits in orthotopic vs. flank models. Despite these dosing adjustments, combination therapy was associated with lower tumor burden and improved survival, compared to the vehicle group. Addressing these tolerability challenges through staggered dosing, reduced individual drug doses, or alternative formulations may enhance the feasibility and efficacy of combination therapy. In patients, the phase 1 FRAME trial reported that intermittent dosing schedules improved tolerability of avutometinib-defactinib combination.^29^ In the phase 2 RAMP 202 clinical trial (NCT04620330), which evaluated avutometinib ± defactinib in recurrent KRAS G12-mutant non-small cell lung cancer (NSCLC), the combination regimen demonstrated a manageable safety profile with no new safety issues identified.^35^ Systemic toxicities included dermatologic events (rash), gastrointestinal effects (diarrhea and nausea), and creatine phosphokinase that were commonly reported in clinical study with KRAS-mutant NSCLC treated with these agents.^35^ The use of avutometinib, by blocking both RAF and MEK signaling without the compensatory MEK activation seen with MEK-only inhibitors, has supported clinical investigation in RAS pathway-driven cancers such as KRAS-mutant NSCLC (NCT04620330). More encouraging activity has been observed in other settings like recurrent low-grade serous ovarian cancer.^35^

### Clinical Implications and Future Directions

Avutometinib demonstrated robust efficacy in multiple preclinical models, highlighting its ability to target MAPK-driven tumor growth effectively. However, since only a small subset of meningiomas harbor MAPK pathway activation,^13^ further investigation is needed to determine the optimal genetic context for response to avutometinib in meningioma. Exploration of avutometinib monotherapy for biomarker-driven clinical trials, specifically on the rare subset of patients with meningioma harboring MAPK pathway activation, may have translational impact. FAK inhibition with defactinib, on the other hand, showed *in vitro* activity in non-MAPK-driven patient-derived cell lines. In the context of clinical trial data showing FAK inhibition with encouraging activity in patients with NF2-mutant meningiomas,^10^ defactinib also warrants additional investigation in *in vivo* models. However, the right biomarkers of response still need to be worked out.

One limitation of our study is the reliance on IOMM-Lee and CH157-MN models in *in vivo* studies. While these are valid meningioma models, MAPK mutations are very rare in meningiomas and the available meningioma cell lines do not fully capture the broad genomic and phenotypic diversity of meningiomas. Expanding future studies to include a wider range of meningioma genotypes, particularly those with clinically relevant mutations beyond *NF2*, will be essential for identifying biomarkers of response and resistance to the therapies under investigation. Additionally, functional genomic approaches such as target knockdown or overexpression were not used to formally confirm drug specificity in our models. Genetic validation of target dependency is an important future direction.^36^ Despite the rationale for dual FAK and MAPK inhibition, the observed tolerability challenges highlight the need for optimization. Future and ongoing studies include establishing patient-derived intracranial tumor models and organoid models, to evaluate these strategies in clinically relevant settings. By incorporating models that better reflect the heterogeneity of meningiomas, including *NF2*-wildtype and non-MAPK-driven tumors, we can refine our understanding of which patient subgroups may derive the greatest benefit from targeted therapies and ultimately improve outcomes for this challenging patient population.

## Supplementary Material

vdag210_Supplementary_Data

## Data Availability

The data generated in this study is available upon request from the corresponding authors.
