# Supplementary material for: Combination treatment with RAF/MEK inhibitor avutometinib and FAK inhibitor defactinib in preclinical models of meningioma
Source: Neurooncol Adv. 2026 Aug 12;8(1):vdag210. doi: 10.1093/noajnl/vdag210 (PMC13629435; doi:10.1093/noajnl/vdag210)
Supplement: Supplementary file 1 [file vdag210_supplementary_data.zip › (06.17.2026) Supplementary Methods.docx]

**Supplementary Methods

Snapshot Profiling**

This test utilizes Anchored Multiplex PCR (AMP) for single nucleotide variant (SNV), insertion/deletion (indel), and copy number detection in genomic DNA using the ArcherDx platform and Illumina NextSeq next-generation sequencing (NGS). Briefly, a board-certified molecular pathologist performed microscopic review of routine H&E-stained sections using validated digital pathology or traditional microscopic workflows. The appropriate region(s) of interest were identified and circled, followed by tumor enrichment via macrodissection prior to nucleic acid extraction. The ArcherDx VariantPlex Pan Solid Tumor v2 protocol was used to target the coding sequence of genes listed below. Illumina NextSeq 2×150 base paired-end sequencing reads were demultiplexed to generate FASTQ files that were transferred to the Archer Analysis pipeline for read alignment to the hg19 human genome, variant detection, and annotation. This test has been validated to detect (1) SNV and indel variants at 5% allelic frequency or higher in target regions with sufficient read coverage, (2) gene-level copy number variants, and (3) an approximated tumor mutation burden (TMB).

TMB was generated after standard DNA somatic variant analysis, filtering, counting, and fitting based on the ArcherDx TMB model to provide a score that correlates with TMB as measured by whole exome sequencing (WES), reported as mutations per megabase (Mb). A summarized TMB category (high, intermediate, or low) is also provided. Due to variability in panel-based TMB calculations, an intermediate range of 5–20 mutations/Mb is adopted between the low and high categories. TMB estimation based on variant counting in targeted panels is an approximation of tumor mutation burden compared to other assays (e.g., WES).

**Western blot analysis**

Cells were cultured in medium containing 2% Matrigel. Protein lysates were separated by NuPAGE™ 4-12% Bis-Tris Protein Gels electrophoresis (Invitrogen) and transferred to PVDF membranes (MilliporeSigma). Membranes were blocked in 0.2% BSA in TBST, incubated overnight with primary antibodies, and subsequently with HRP-conjugated secondary antibodies. Immunoreactive bands were developed with ECL substrate reagent (Thermo Fisher) and detected using an Azure Biosystems c600. Primary antibodies: phospho-ERK (Thr202/Tyr204, CST 9101, RRID:AB_331646), ERK (CST 9102, RRID:AB_330744), phospho-FAK (Tyr397, CST 3283, RRID:AB_2173659), FAK (CST 13009, RRID:AB_2798086), NF2 (CST 12888, RRID:AB_2650551), GAPDH (Santa Cruz 25778, RRID:AB_10167668).

For western blot of tumor tissue, snap-frozen tissue (~100 mg, range 40–160 mg) was homogenized in 500 µL Pierce RIPA lysis buffer (Cat# 89900, Thermo Scientific) with 1X Halt Protease and Phosphatase Inhibitor cocktails (Cat# 87786 and 78420, Thermo Scientific), incubated for 30 minutes at 4°C with agitation, and sonicated at 40% amplitude for 30 seconds (1 second on/2 seconds off). Supernatant was collected after centrifugation at 10,000g for 10 minutes at 4°C and quantified using Pierce BCA Protein Assay Kit (Cat# 23225, Thermo Scientific).

**Western blot quantification**

Densitometric analysis of Western blot bands was performed using ImageJ software (NIH). Blot images were converted to 8-bit grayscale, and individual bands were selected using the rectangular selection tool. The integrated density (area × mean gray value) was measured for each band, and background signal was subtracted. Protein expression levels were normalized to the corresponding loading control (e.g., β-actin), and relative intensities were reported as fold-change compared to control.

***In vivo* pharmacokinetics (PK) and pharmacodynamics (PD) study**

To evaluate pharmacokinetics and pharmacodynamics, mice bearing IOMM-Lee-FmC (IOMM-Lee cells stably expressing firefly luciferase and mCherry) intracranial tumors were treated with oral doses of VS-4718 (50 mg/kg, twice a day), avutometinib (0.3 mg/kg, once a day), or their combination for three consecutive days. Treatment was initiated approximately 21 days post-implantation, and the study included four groups: vehicle control (n=5), VS-4718 alone (n=5), avutometinib alone (n=5), and combination therapy (n=5).

Tumors and whole blood were collected three hours after the final dose for all groups. Plasma was separated from blood samples collected in K2-EDTA tubes. Intracranial tumors were excised, weighed, flash-frozen, and stored at –80°C. Both plasma and tumor samples were harvested, frozen immediately, and stored for further analysis. Plasma and tumor samples were subsequently shipped to Charles River for pharmacokinetic analysis, where plasma or biological samples (10 µL each) were extracted using protein precipitation. Each sample was mixed with 60 µL of acetonitrile containing the internal standard (Carbamazepine), briefly vortexed, and centrifuged at 3000 rpm for 5 minutes. A 50 µL aliquot of the resulting supernatant was transferred to a clean plate and diluted with 50 µL of Milli-Q water prior to LC-MS/MS analysis. Chromatographic separation was performed on a Waters XBridge C18 column maintained at 45 °C, with mobile phases consisting of 95:5:0.1 Water:Acetonitrile:Formic Acid (phase A) and 50:50:0.1 Methanol:Acetonitrile:Formic Acid (phase B). Detection was performed using a SCIEX API 5500 mass spectrometer equipped with an electrospray ionization (ESI) source operating in positive ion mode at 550 °C.

**Bioluminescent Imaging (BLI)**

**In vivo bioluminescent imaging (BLI)** at Mayo Clinic: Images were captured 10 minutes post-injection on an IVIS 2.50 cooled CCD camera (Xenogen 200 series) using Living Image software. Brain ROIs were defined using a fixed-area circle positioned according to BLI signal in the target region, guided by anatomical reference photographs. BLI images were overlaid onto corresponding white-light images for anatomical reference. ROI analysis was performed using AURA software (Spectral Instrument Imaging).

At MGH, BLI was performed on a Spectral Instruments Imaging AMI-X system (Tucson, AZ). Mice received 300 µL of D-Luciferin potassium salt (15 mg/mL, i.p.) and were anesthetized with 2% isoflurane in oxygen throughout imaging. Sequential acquisitions of 60 s and 0.5 s across all binning groups were obtained beginning 5 minutes post-injection until peak radiance was reached.

For IOMM-Lee-FmC cells, images were analyzed in AURA software using identical ellipsoidal ROIs around the head of the mouse across all time points, with consistent binning and exposure time, alongside a background ROI. Peak radiance and total photons per second minus background were extracted from sequential acquisitions using a custom Python script, excluding overexposed images.
